# Comparative study on the effects of type 1 and type 2 diabetes on structural changes and hormonal output of the adrenal cortex in male Wistar rats

**DOI:** 10.1186/2251-6581-12-9

**Published:** 2013-01-28

**Authors:** Zohreh Elahi-Moghaddam, Morteza Behnam-Rassouli, Naser Mahdavi-Shahri, Roya Hajinejad-Boshroue, Elaheh Khajouee

**Affiliations:** 1 Biology Department, Faculty of Sciences, Islamic Azad University of Mashhad, Mashhad, Iran; 2 Biology Department, Faculty of Sciences, Ferdowsi University of Mashhad, Mashhad, Iran

**Keywords:** Type 1 diabetes, Type 2 diabetes, Adrenal, Rat

## Abstract

**Introduction:**

Diabetes is one of the most common endocrine disorders characterized by hyperglycemia due to defects in insulin secretion, insulin function, or both. Causing dysfunction in the body general metabolism, diabetes-induced chronic hyperglycemia leads to alterations in those endocrine glands involved in regulating the body metabolism. In this line, the present study has been conducted to investigate the effects of type 1 and type 2 diabetes on the structural changes and hormonal output of the adrenal cortex in male Wistar rat.

**Methods:**

Eighteen male Wistar rats were divided into three groups including control, experimental type 1 diabetes (subcutaneous injection of 135 mg/kg alloxan) and experimental type 2 diabetes (8 weeks treatment with drinking water containing 10% fructose). Two months after the induction of both types of diabetes, the level of blood biochemical factors (glucose, insulin, cortisol, triglycerides, cholesterol, LDL, and HDL) were measured. Structural changes of the adrenal cortex were then evaluated, using stereological techniques.

**Results:**

Serum biochemical analysis showed significant difference in the levels of glucose, triglycerides, insulin and cortisol in experimental groups, compared to the control. The results of structural alterations were also indicative of increase in adrenal cortex volume in both types of diabetes.

**Conclusion:**

Probably through increasing HPA axis activity, type1 diabetes-induced hyperglycemia leads to adrenal hypertrophy and increase the hormonal output of adrenal gland.

## Introduction

Diabetes is characterized by chronic hyperglycemia and impaired metabolism of carbohydrates, fats and proteins. The disease is caused by defects in insulin secretion, insulin function, or both. High blood glucose, as the main cause of short-term and late complications of diabetes, leaves serious adverse effects on all body systems including the endocrine system [1]. Through changing the hormonal output of endocrine glands, diabetes contributes to the development of secondary metabolic disorders [2]. On the other hand, neuropathy as the most common neurological complication of diabetes deteriorates the disease condition via affecting the endocrine system, in addition to peripheral and autonomic nervous systems [3]. In patients with diabetes, autonomic nervous system imbalance leads to increased activity of the hypothalamic-pituitary-adrenal (HPA) axis, and consequently hypercortisolism and adrenocortical growth. These alterations are probably due to reduced relative feedback sensitivity to glucocorticoids in different parts of the axis, changes in 11- beta hydroxysteroid dehydrogenase (11B-HSD) enzyme activity, and increased expression of corticotropin-releasing hormone in hypothalamus [4]. In this regard, it has been shown that there is an increase in cortisol secretion and adrenocortical hypertrophy in type 2 diabetic patients who suffer parasympathetic neuropathy, compared with type 1 diabetics with sympathetic neuropathy [5]. Thus, it seems that the degree of HPA axis dysfunction in diabetic patients is associated with the damage of neuronal pathway of the HPA axis and weakening response of glucocorticoids negative feedback [6]. On this basis, changing level of hormones such as insulin and glucocorticoids leads to increased fatty acid release from adipocytes, increased lipoprotein synthesis in liver cells and subsequent occurrence of fatty liver and finally resulting in impaired intracellular insulin signaling and insulin resistance [7]. Thus, changing level of insulin and glucocorticoids, as well as decreased adipocytes sensitivity to insulin are probably the reasons behind elevated levels of blood lipid [7,8]. In addition, via inducing increased expression of some genes in target tissues, inhibition of beta-oxidation of lipids in mitochondria, and increased activity of lipogenic enzymes, glucocorticoids can augment the level of blood fats; alterations that are inhibited by adrenalectomy [2,9]. Insulin inhibits the insulin-sensitive lipase in adipose tissue while in the absence of insulin and glucocorticoids stimulation, increased activity of this enzyme may lead to elevated plasma fatty acids [9]. In the present study, the effects of hyperglycemia, induced by experimental type 1 and type 2 diabetes on the structural changes of the adrenal cortex, the levels of blood biochemical factors, as well as cortisol and insulin in male Wistar rat have been studied.

## Materials and methods

In this study, 18 male Wistar rats, two months of age, were used which were kept, based on Local Animal Ethic Committee protocols, under the standard conditions at 20 ± 2°C temperature and 12:12 light–dark hours with free access to water and standard food. Rats were randomly divided into 3 groups (n = 6) including control, experimental type 1 and type 2 diabetes. For the induction of type 2 diabetes and insulin resistance, animals were fed with drinking water containing 10% fructose (Merk, Germany) for two months (i.e. until 4 months of age) [10,11]. During this period, control and type 1 diabetic group were fed with standard food and water.

To ensure the induction of type 2 diabetes, rats were given glucose tolerance test, and fasting insulin resistance index (FIRI) were compared between control and type 2 diabetic groups. FIRI was calculated via blood sampling from ocular sinus and measurements of fasting insulin and glucose levels. To keep the stable state of insulin resistance, fructose treatment was continued during the experimental period (the next eight weeks). Animals’ blood glucose changes were regularly measured during the experiment.

### Glucose tolerance test

At the end of the eighth week of fructose treatment and after 12 hours fasting, in addition to evaluating the body weight, fasting blood glucose was measured using a glucometer. Then, 40% glucose solution with the concentration of two grams per kilogram of body weight was intraperitoneally injected to rats, and blood glucose level was evaluated at 30, 60 and 120 minutes after glucose injection.

### FIRI calculation

Fasting insulin resistance index is a criterion to determine the induction of type 2 diabetes and can be obtained through the following formula [12]:

$$\mathrm{FIRI}=\frac{\mathrm{Fasting}\;\mathrm{insulin}\left(\frac{\mathrm{mIU}}{\mathrm{ml}}\right)\times \mathrm{Fasting}\;\mathrm{glucose}\left(\frac{\mathrm{mg}}{\mathrm{dl}}\right)}{25}$$

Type 1 diabetes was induced by a single subcutaneous injection of 135 mg/kg body weight alloxan (Merk, Germany) to four-month-old rats. Three days after the injection, blood fasting glucose was measured to confirm the induction of type 1 diabetes.

At the end of the experimental period (about six months of age), blood samples were collected from all the study groups and serum biochemical factors (glucose, triglycerides, cholesterol, cholesterol-LDL and cholesterol-HDL), as well as insulin and cortisol were measured.

### Preparation and selection of tissue samples

#### Cavalier technique

After perfusion, removal of the adrenal gland, and additional fixation, one of the adrenal glands of each animal was randomly selected for tissue processing. The paraffin blocks were then serially sectioned at 7 microns thick. Initial sections were excluded, and adrenal sections were serially selected 3 per each 30 sections; since sampling was initiated by a number between 1 and 30, e.g. 15, the first series included sections number 15-17, second series 45-47 and third series 75-77 and so on. The aim of this approach was to possess all parts of the adrenal gland in the total samples of each group. After Hematoxylin-Eosin staining, each section was photographed by a digital camera (Olympus Dp71). To provide a complete image of the adrenal gland, the first section in each series of sections were photographed by 2x objective lens. The prepared images were then used for determining the adrenal gland volume, using Cavalieri's principle.

Cavalieri's principle is widely used to estimate the reference volume in stereological studies, especially biomedical evaluations. In this technique, the volume of the considered structure can be measured using a series of random sections with equal distances from one another [13].

**Figure 1:**
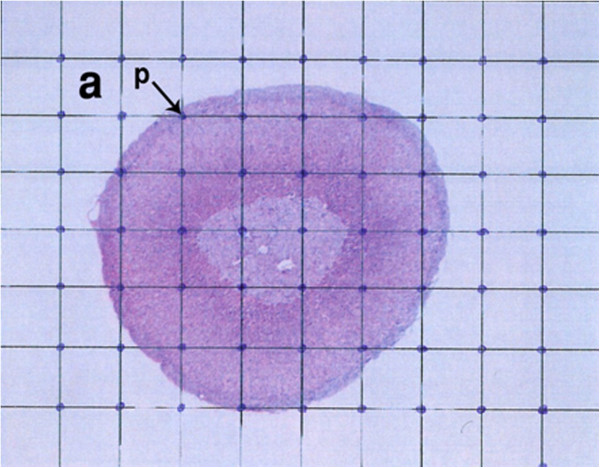
In Cavalieri's principle, the number of points on the considered surface area is counted.

Various stages of this procedure are summarized as follows;

The sample (adrenal gland in this study) was sectioned as parallel systematic sections with equal distance (d). Sectioning was initiated randomly. A transparency paper with a number of uniform points with equal distance (Figure 1) was then placed on sections, and the number of points on the considered cross-section (adrenal cortex) was counted; thus, the total surface area of the adrenal gland (ΣA) on all sections was calculated, and total volume of the adrenal cortex was estimated through the following formula:

$$V_{\left(\mathrm{Adrenal}\;\mathrm{gland}\right)}=d\times \sum A$$

V = total volume of the considered object (mm^3^)

d = distance between sections (mm)

ΣA = total surface area of the adrenal gland which is obtained by the following formula:

$$\sum A=\sum p\times a\left(p\right)$$

Σ p = number of points counted on the whole surface areas of the sample

a (p) = area associated with each point (p) (mm^2^).

Thus: V = d × ∑ p × a(p)

### Statistical analysis

To determine the significant difference between different groups, one-way analysis of variance (ANOVA) was used in SPSS_15_ statistical software. Significant differences between the two groups were calculated using student *t*-test analysis (P <0.05 was considered statistically significant). Figures and tables were also drawn by Excel software.

## Results

**Figure 2:**
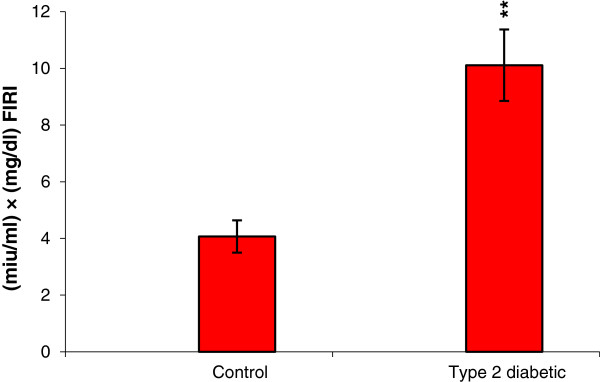
**Comparison of FIRI between control and type 2 diabetic animals.** *p <0.05, **p <0.01 control group compared with type 2 diabetes. Sample size: 6 male adult rats. Statistical analysis: Student *t*-test. Type of study: experimental.

**Figure 3:**
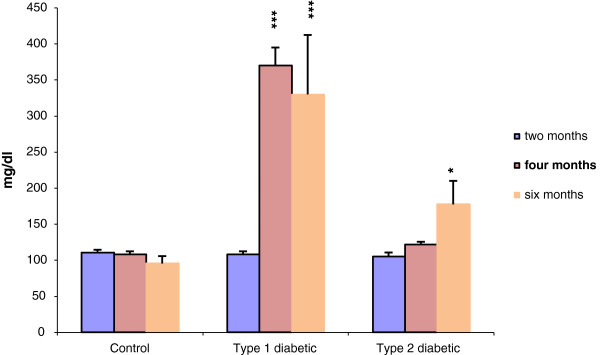
**Changes in serum glucose level in all the study groups during the experimental period.** *p <0.05, **p <, 0.01, ***p <0.001 control group compared with diabetic groups. Sample size: 6 male adult rats. Statistical analysis: ANOVA and student *t*-test. Type of study: experimental.

**Figure 4:**
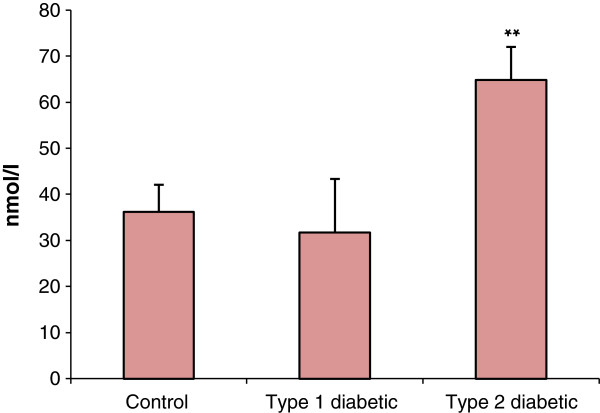
**Comparison of cortisol levels between control and diabetic groups.** *p <0.05, **p <0.01 diabetic groups compared with control. Sample size: 6 male adult rats. Statistical analysis: ANOVA and student *t*-test. Type of study: experimental.

The results of glucose tolerance test showed that fasting blood glucose was significantly different at 30 min and 2 h after glucose injection between control and type 2 diabetic groups. FIRI also revealed a significant increase in type 2 diabetic group compared with control (Figure 2). In the control and experimental groups, the level of blood glucose was measured at the beginning (two months of age), after induction of type 1 and type 2 diabetes (four months of age) and the end of the study (six months of age) (Figure 3). Changes in levels of other blood biochemical factors (Table 1), as well as cortisol (Figure 4) were also evaluated.

**Table 1:** The mean glucose, cortisol, insulin, triglyceride, cholesterol, cholesterol LDL, and cholesterol HDL in control and experimental groups at the end of study

	**Control**	**Type 1diabetes**	**Type 2 diabetes**
**Serum Glucose Level (mg/dl)**	95.5 ± 10.27	330 ± 82.55***	177.5 ± 32.6*
**Cortisol (nmol/l**	36.2 ± 5.88	31.74 ± 11.6	64.8 ± 7.19 **
**Insulin (miu/ml)**	1.07 ± 0.11	0.97 ± 0.08	1.52 ± 0.17*
**Triglyceride (mg/dl)**	50.75 ± 4.09	62 ± 10.64*	68.25 ± 16.02
**Cholesterol (mg/dl)**	61.25 ± 2.29	69.5 ± 4.15	61. 25 ± 1.25
**LDL (mg/dl)**	36.25 ± 1.8	30.7 ± 3.61	35 ± 1.82
**HDL (mg/dl)**	16.33 ± 1.2	15.5 ± 1.41	15.33 ± 0.49

*p < 0.05 **p < 0.01 comparison (Mean ± SEM) with control group.

Sample size: 6 male rats.

Statistical methods: ANOVA and student *t*-test.

Type of study: Experimental.

**Figure 5:**
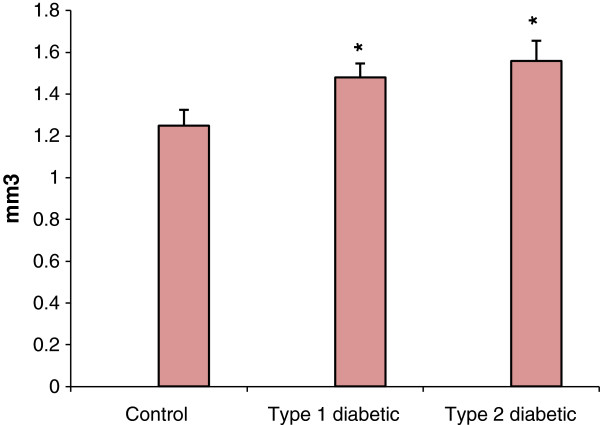
**Evaluation of the adrenal cortex volume (mm**^**3**^**) in type 1 and type 2 diabetic rats compared with control.** *p <0.05, **p <0.01 diabetic groups compared with control. Sample size: 6 male adult rats. Statistical analysis: ANOVA and student *t*-test. Type of study: experimental.

The results achieved from the measurement of adrenal cortex volume displayed a remarkable increase in type 1 and type 2 diabetic groups in comparison with control (Figure 5).

## Discussion

The results of this study showed the induction of type 2 diabetes and insulin resistance following 8 weeks treatment with fructose. The significant increases in FIRI and glucose are well indicative of this finding (Figure 2 and 3). In type 1 diabetic group, increase in blood glucose, appetite, urination, thirst during experimental period indicate the induction of type I diabetes.

The results in the measurement of adrenal cortex volume, using Cavalieri's principle demonstrated a remarkable increase (p < 0.05) in the volume of adrenal cortex in diabetic, compared with control group; however, the increase was more significant in type 2 than type 1 diabetes (Figure 5). Such an increment in the adrenal cortex volume in diabetic groups is in consistence with the results of previous studies [14-16]. Hypertrophy and increased adrenal cortex volume can cause increased secretion of adrenal hormones, including cortisol. In this regard, measurement of serum cortisol levels showed significant increase (p <0.01) in serum cortisol levels in type 2 diabetic group (but not in type 1 diabetes) (Table 1). In this line, assessment of urinary metabolites of cortisol has shown decreased level of these metabolites in the urine of patients with type 1 diabetes; such a decrease is probably due to the impairment of metabolic pathways in hyperglycemic condition, such as 11-beta-HSD pathway, which converts cortisol to its inactive metabolite (cortisone) [17]. On the other hand, circadian measurement of cortisol and ACTH levels in 170 patients with type 2 diabetes revealed significantly increased in urinary secretion of cortisol as well as HPA axis activity [18]. These findings indicate that under hyperglycemic condition, especially type 2 diabetes, the HPA axis activity and consequently hormonal output of the adrenal cortex is affected. This may lead to the incidence of secondary dysfunction in the activity of other endocrine glands and subsequently exacerbation and complication of disease if no control of blood glucose is achieved [4].

Although in physiological condition, HPA axis activity is influenced by negative feedback of glucocorticoids [19], chronic glucocorticoids increase reduces the sensitivity of this axis to feedback effects of these hormones. Such a reduced sensitivity may partly responsible for the functional impairments and increased activity of HPA axis in hyperglycemic conditions [19,20]. It is apparently that increase in ACTH levels contributes to strong stimulation of adrenal cortex, hypertrophy and elevated hormonal output [21]. Impaired lipid metabolism and insulin resistance may also be other possible causes of increased volume of adrenal cortex and cortisol secretion [21,22]. In this respect, the results obtained by measurement of blood biochemical factors show significantly high serum triglyceride levels in diabetic rats (Table 1). These findings strongly emphasize the interaction between the two metabolic hormones, cortisol and insulin [2,21]. Therefore, this is not unexpected that dysfunction in one causes dysfunction in another. Similar to the results obtained from the present study (Table 1), previous reports showed that type 1 and type 2 diabetes-induced hyperglycemia augment the levels of cholesterol, triglycerides, LDL and VLDL, and diminishes the level of HDL [23-26]. Although such significant and adverse changes in blood lipids and lipoproteins can be ascribed to alterations in the serum level of hormones such as insulin and cortisol, the possibility of other reasons such as liver dysfunction [27] and subsequent alteration in liver enzymes level [28] cannot be ignored.

## Competing interest

The authors declare that they have no competing interests.

## Authors’ contributions

ZM drafted the manuscript. All authors read and approved the final manuscript. Thank you very much for your reply to advise me to improve the quality of revised manuscript.
